# Clinical characteristics and prediction analysis of pediatric urinary tract infections caused by gram-positive bacteria

**DOI:** 10.1038/s41598-021-90535-6

**Published:** 2021-05-26

**Authors:** Yu-Lung Hsu, Shih-Ni Chang, Che-Chen Lin, Hsiao-Chuan Lin, Huan-Cheng Lai, Chin-Chi Kuo, Kao-Pin Hwang, Hsiu-Yin Chiang

**Affiliations:** 1 Division of Infectious Diseases, China Medical University Children’s Hospital, China Medical University, No. 2 Yuder Rd., North Dist., Taichung, 404 Taiwan, ROC; 2 Big Data Center, China Medical University Hospital, No. 2 Yuder Rd., North Dist., Taichung , 404 Taiwan, ROC; 3 Kidney Institute, China Medical University Hospital, Taichung, 404 Taiwan, ROC; 4 Department of Medical Research, China Medical University Hospital, Taichung, 404 Taiwan, ROC

**Keywords:** Bacterial infection, Urinary tract infection, Risk factors, Infectious-disease epidemiology

## Abstract

Gram-positive (GP) pathogens are less accounted for in pediatric urinary tract infection (UTI), and their clinical impact is underrecognized. This study aimed to identify predictors of GP uropathogens in pediatric UTI. In this 14-year retrospective cohort of pediatric patients with UTI, we classified first-time UTIs cases into those caused by GP or Gram-negative (GN) bacteria. We constructed a multivariable logistic regression model to predict GP UTI. We evaluated model performance through calibration and discrimination plots. We developed a nomogram to predict GP UTI that is clinically feasible. Of 3783 children with first-time UTI, 166 (4.4%) were infected by GP and 3617 (95.6%) by GN bacteria. Among children with GP UTI, the most common uropathogens were vancomycin-resistant *Enterococcus faecalis* (VRE) (27.1%), *Staphylococcus saprophyticus* (26.5%), and coagulase-negative *Staphylococci* (12.7%). Eight independent risk factors were associated with GP UTI: Age ≥ 24 months (odds ratio [OR]: 3.21), no prior antibiotic use (OR: 3.13), serum white blood cell (WBC) count < 14.4 × 10^3^/μL (OR: 2.19), high sensitivity C-reactive protein (hsCRP) < 3.4 mg/dL (OR: 2.18), hemoglobin ≥ 11.3 g/dL (OR: 1.90), negative urine leukocyte esterase (OR: 3.19), negative urine nitrite (OR: 4.13), and urine WBC < 420/μL (OR: 2.37). The model exhibited good discrimination (C-statistic 0.879; 95% CI 0.845–0.913) and calibration performance. VR *E. faecalis*, the leading GP uropathogen causing pediatric UTI, requires early detection for infection control. Our model for predicting GP UTI can help clinicians detect GP uropathogens and administer antibiotic regimen early.

## Introduction

Urinary tract infection (UTI) is a leading diagnosis in pediatric patients in the United States and entailed hospital charges exceeding US$520 million in 2006^1^. Significantly high morbidity and the subsequent medical sequelae (e.g., renal scarring or impaired kidney function) are associated with pediatric UTIs, especially in children younger than 2 years of age^2^. Early appropriate antibiotic treatment prevents morbidity and reduces long-term sequelae^3^. Therefore, better prediction of the offending pathogen in pediatric UTI can help in prescribing empirical antibiotic therapy to improve prognosis.

*E. coli* accounts for 74% of UTIs, followed by *Klebsiella* species (8.8%), in infants^4^. Empirical antimicrobial therapy eradicates the predominant Gram-negative (GN) bacteria in pediatric UTI. However, Gram-positive (GP) bacteria, such as *Enterococcus* spp., *Streptococcus* spp., or *Staphylococcus* spp. (e.g., *S. aureus* and *S. saprophyticus*) are less common uropathogens, with proportion ranging from 1.0 to 6.3%^4^. GP bacteria have not received much attention in the primary care setting^5,6^ and are considered contaminants, despite the growing contrary evidence^7,8^. Moreover, pediatric patients with UTIs caused by GP bacteria, particularly *Enterococcus* spp. and *S. aureus*, are likely to have concomitant anatomical abnormalities, such as hydronephrosis or vesicoureteral reflux (VUR), and do not respond to empirical antibiotic therapy for GN bacterial infection^7–10^.

No systematic analysis has compared clinical features and outcomes between GN and GP UTI. To fill this knowledge gap, we conducted a retrospective study to compare the clinical features and outcomes of GN and GP UTIs and developed a prediction model for GP UTIs in patients younger than 18 years.

## Methods

### Data source

This retrospective cohort study was conducted at China Medical University Hospital (CMUH), a tertiary medical center in central Taiwan. The data were sourced from CMUH-Clinical Research Data Repository (CRDR), which accumulates the electronic health records (EHR) of the single unified views of 2,660,472 patients who sought care at CMUH between 2003 and 2016^11,12^. Patient information included data on administration and demography, diagnoses, medical and surgical procedures, prescriptions, laboratory measurements, physiological monitoring data, hospitalization, and catastrophic illness status as defined by the National Health Insurance Administration. This study was approved by the Big Data Center of CMUH and the Institutional Review Board of CMUH (105-REC3-068), with a waiver regarding informed consent. All methods of this study were performed in accordance with the relevant guidelines and regulations.

### Study population

**Figure 1:**
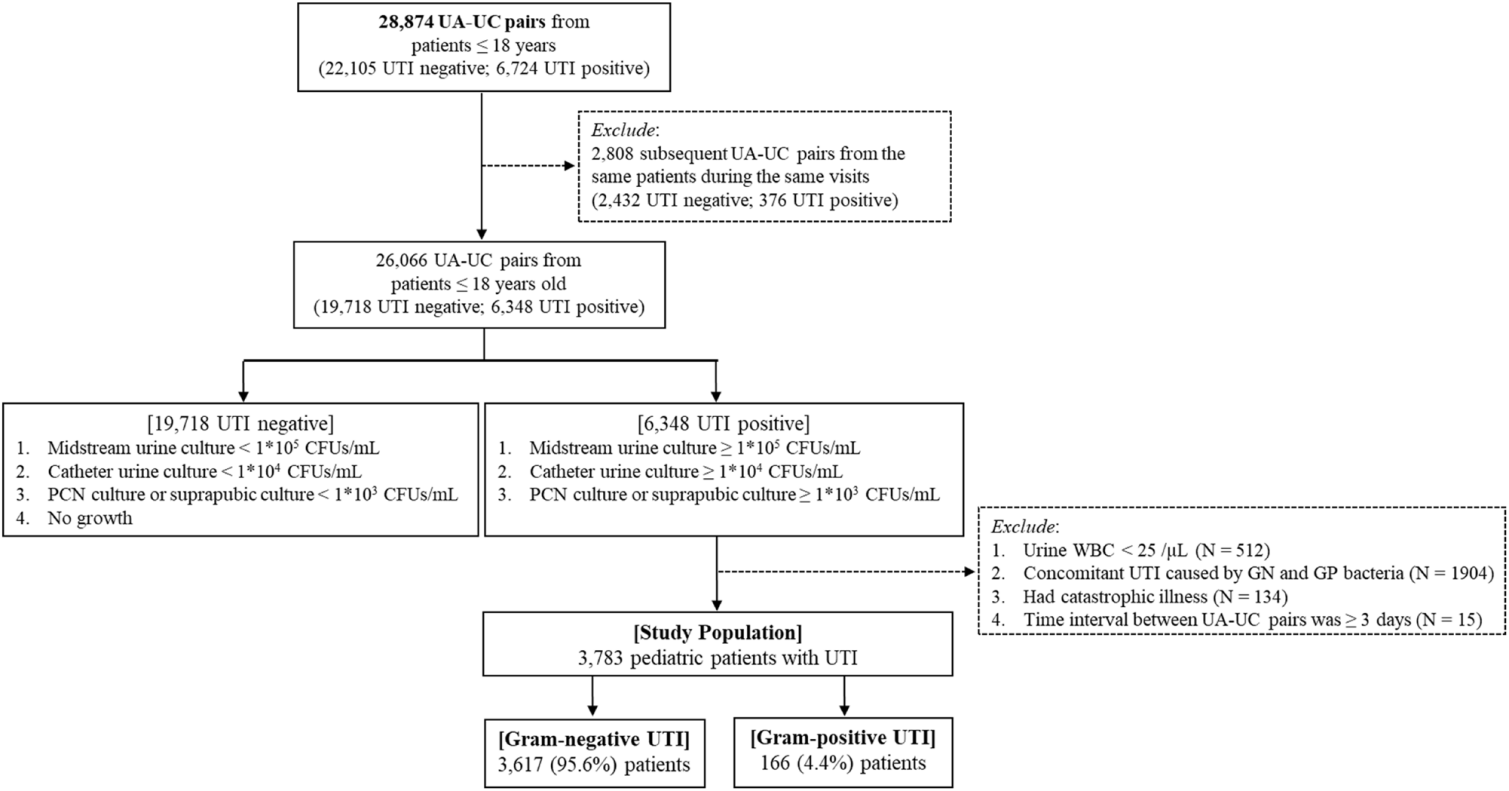
Flowchart of the selection process of the study population (N = 3783 patients).

From 2003 through 2016, we identified 28,874 paired urinalysis (UA) and urine culture (UC) samples obtained from pediatric patients (age ≤ 18 years) at CMUH, and 26,066 UA–UC pairs were obtained for the same visit from the same patient^13^ (Fig. 1). One UA was paired with one UC, which was ordered within 7 days after and closest to the time of UA. In Taiwan, the physician’s order of UA and UC exam is supervised by the National Health Insurance (NHI) Administration and can only be reimbursed if the UA and UC exam follow symptoms of UTI (e.g., fever, dysuria, urgency, frequency, incontinence, abdominal, back, or flank pain, nausea, vomiting, poor feeding, irritability, jaundice, or weight loss)^14^.

We classified paired UA–UC cases into positive for UTI (N = 6348) and negative for UTI (N = 19,718) based on sampling source-specific cutoffs of colony forming units (CFUs). UTI is defined as urine culture containing ≥ 10^5^ CFU/mL in a midstream urine specimen, ≥ 10^4^ CFU/mL in a catheter urine specimen, or ≥ 10^3^ CFU/mL in a percutaneous nephrostomy (PCN) or suprapubic urine specimen, based on the EAU/ESPU guidelines^15^. The unclassified urine cultures, including those with no growth or with contamination (i.e., presence of more than three organisms) were classified as negative for UTI. Moreover, children included as UTI positive all received antibiotics and the antibiotic treatment for inpatients were routinely approved by pediatric infectious disease specialist and reviewed by the NHI Administration for reimbursement.

To compare the characteristics between patients with GP and GN pathogens, our study population was formed from 6348 UTI positive cases and divided into UTI caused by GP or GN pathogens. In addition, we excluded patients if their urine WBC count was less than 25/μL, if their urine culture grew both GP and GN pathogens, if they had catastrophic illness as defined by the Ministry of Health and Welfare, Taiwan^16^, and if the time interval between UA and UC pairs was ≥ 3 days. Our study population, consisting of 3783 children with UTIs—3617 with GN UTI and 166 with GP UTI—were used in all subsequent analyses.

### Covariates

The details of urinalysis and urine culture methods are described in “Supplemental Text”. Prematurity was defined using the ICD-9 codes of 765.xx that were presented in the EHR prior to the UA order. Hydronephrosis (ICD-9 codes 591.xx), vesicoureteral reflux (ICD-9 code 593.7x), and hypospadias (ICD-9 code 752.6x) were defined using ICD-9 codes presented in the EHR within 1 year prior to the UA order. Results from imaging studies, such as kidney sonography, voiding cystoureterography, and Tc 99 m-dimercaptosuccinic acid renal scintigraphy, performed within 1 year prior to the UA order, were evaluated. History of Foley catheterization was defined as placement of Foley catheter within 3 months prior to the UA order. History of bacteremia was defined as having positive blood culture within 3 months prior to the UA order. Serum biochemistry profiles for WBC, Hb, platelets, and high sensitivity C-reactive protein (hsCRP), which were measured within 7 days prior to the UA order, were analyzed. Continuous variables were dichotomized using the median value as the cutoff.

### Outcome variables

Length of stay (LOS) was defined as the duration between admission and discharge for patients who were admitted to the CMUH. Recurrent UTI was defined as having ≥ 2 UTIs within 6 months or having ≥ 3 UTIs within 1 year following the index UA. Bacteremia was defined as having positive blood culture within 90 days following the index UA order.

### Statistical analysis

Descriptive statistics are presented as mean (standard deviation) and median (inter-quartile range) for continuous variables and as frequency and proportion (%) for categorical variables. We compared characteristics between patients with UTI caused by GP and GN bacteria by using the Wilcoxon rank-sum test or the chi-square test. We established a multivariable logistic regression model using statistically significant or clinically relevant variables to predict GP UTI. We examined the discrimination and calibration performance of the model by using the c-statistic, the receiver-operating curve, and the calibration plot^17^. To maximize clinical utilization, we calculated the risk points on the basis of risk estimate and developed a nomogram by using R with the rms package^18^. Decision curve analysis was used to evaluate the clinical net benefit of our prediction model^19^. All analyses were performed using SAS Version 9.4 (Cary, NC, USA. https://www.sas.com) or R Version 3.5.1 (R Foundation for Statistical Computing, Vienna, Austria. https://www.r-project.org). The significance level was set at 0.05, and all tests were two-tailed.

## Results

Of 3783 cases with first-time UTI, 166 (4.4%) were caused by GP and 3617 (95.6%) by GN bacteria (Fig. 1; Table 1). The proportion of pediatric UTI caused by GP bacteria remained stable from 2003 (6.0%) through 2016 (4.7%; Supplemental Fig. 1). The most common GP bacteria included vancomycin-resistant *E. faecalis* (27.1%), *S. saprophyticus* (26.5%), coagulase-negative *Staphylococci* (12.7%), *S. agalactiae* group B (8.4%), and *Enterococcus spp.* (4.8%), whereas the most common GN bacteria included *E. coli* (79.7%), *E. coli*-ESBL (8.0%), *P. mirabilis* (3.3%), *Klebsiella pneumoniae* (2.9%), and *Citrobacter koseri* (1.2%; Supplemental Figs. 2 and 3). The predominant GP bacteria varied by age group—VR *E. faecalis* ranked first among children < 12 years old and *S. saprophyticus* ranked first among those ≥ 12 years of age (Supplemental Fig. 4). Most patients with GN UTI received cefazolin (71.0%) and/or gentamycin (50.8%) as the empirical treatment, whereas approximately a third of the patients with GP UTI received cefazolin (36.7%) and/or cephradine (27.7%; Supplemental Table 1).

**Table 1:** Demographic and clinical characteristics of 3783 pediatric patients with urinary tract infection, 2003–2016 at China Medical University Hospital (CMUH), Taiwan.

Variable^a^	Total (N = 3783)	Gram-negative (N = 3617)	Gram-positive (N = 166)	P-value^b^
**Age at UA order (month)**	7.8 (3.7, 42.2)	7.5 (3.7, 35.7)	127 (10.4, 202)	< 0.001
≤ 1 month (28 days)	128 (3.4)	109 (3.0)	19 (11.4)	< 0.001
29–90 days	592 (15.6)	588 (16.3)	4 (2.4)	
91 days–< 6 months	1594 (42.1)	1574 (43.5)	20 (12.0)	
6 months–< 24 months	287 (7.6)	282 (7.8)	5 (3.0)	
≥ 24 months	1182 (31.2)	1064 (29.4)	118 (71.1)	
**Girl**	1758 (46.5)	1657 (45.8)	101 (60.8)	< 0.001
**Index visit status**				0.467
Inpatient	940 (24.8)	904 (25.0)	36 (21.7)	
ER	2243 (59.3)	2137 (59.1)	106 (63.9)	
Outpatient	600 (15.9)	576 (15.9)	24 (14.5)	
**Sample type**				< 0.001
Midstream	2468 (65.2)	2417 (66.8)	51 (30.7)	
Catheter, PCN, or suprapubic	1315 (34.8)	1200 (33.2)	115 (69.3)	
**Comorbidity**				
Prematurity^c^	128 (3.4)	124 (3.4)	4 (2.4)	0.478
Hydronephrosis^d^	65 (1.7)	61 (1.7)	4 (2.4)	0.483
VUR^d^	56 (1.5)	51 (1.4)	5 (3.0)	0.095
Hypospadias^d^	9 (0.2)	8 (0.2)	1 (0.6)	0.324
**Clinical history**				
Antibiotic use^e^	3626 (95.8)	3475 (96.1)	151 (91.0)	0.001
Admission^e^	361 (9.5)	339 (9.4)	22 (13.3)	0.096
Foley catherization^e^	1379 (36.5)	1358 (37.5)	21 (12.7)	< 0.001
Renal echo^d^	436 (11.5)	420 (11.6)	16 (9.6)	0.436
VCUG^d^	83 (2.2)	77 (2.1)	6 (3.6)	0.201
DMSA renal scan^d^	16 (0.4)	14 (0.4)	2 (1.2)	0.112
Bacteremia^e^	292 (7.7)	287 (7.9)	5 (3.0)	0.020
**Fever (> 38 °C) (N = 3140)**	2016 (64.2)	1974 (65.7)	42 (31.1)	< 0.001
**Serum biochemical profiles**^f^				
WBC (10^3^ per μL)	14.4 (10.7, 18.7)	14.6 (10.8, 18.8)	11.1 (8.6, 13.3)	< 0.001
Hemoglobin (g/dL)	11.3 (10.3, 12.2)	11.2 (10.3, 12.2)	12.6 (11.5, 13.6)	< 0.001
Platelet (10^3^ per μL)	341 (264, 431)	344 (266, 432)	272 (240, 365)	< 0.001
hsCRP (mg/dL)	3.4 (1.1, 7.2)	3.5 (1.1, 7.3)	0.7 (0.1, 3.3)	< 0.001
**Urinalysis**				
Bacteria (+)	1907 (50.4)	1861 (51.5)	46 (27.7)	< 0.001
Leukocyte esterase (+)	3459 (91.4)	3342 (92.4)	117 (70.5)	< 0.001
Nitrite (+)	1416 (37.4)	1404 (38.8)	12 (7.2)	< 0.001
pH				0.001
Mean (SD)	6.22 (0.67)	6.21 (0.66)	6.44 (0.75)	< 0.001
Median (Q1–Q3)	6.00 (6.00–6.50)	6.00 (6.00–6.50)	6.50 (6.00–7.00)	< 0.001
4.5–< 5.5	161 (4.3)	157 (4.3)	4 (2.4)	
5.5–< 6.5	2138 (56.5)	2065 (57.1)	73 (44.0)	
6.5–9.0	1482 (39.2)	1393 (38.5)	89 (53.6)	
Specific gravity				
Mean (SD)	1.013 (0.008)	1.013 (0.008)	1.016 (0.008)	< 0.001
Median (Q1, Q3)	1.010 (1.005, 1.019)	1.010 (1.005, 1.018)	1.015 (1.010, 1.020)	< 0.001
**Urine microscopic examination**				
WBC (per μL)	420 (110, 1000)	452 (120, 1000)	96 (45, 346)	< 0.001
RBC (per μL)	22 (7, 80)	22 (8, 80)	16 (6, 69)	0.270
Epithelial cell (per μL)	2 (0, 5)	2 (0, 5)	3.5 (1, 11)	< 0.001
**Outcome**				
Length of hospitalization (day)^g^	5 (4, 8)	5 (4, 8)	4 (3, 5)	0.001
Recurrent UTI^h^	380 (10.0)	369 (10.2)	11 (6.6)	0.134
Ureteroneocystomy^i^	55 (1.5)	49 (1.4)	6 (3.6)	0.017
Bacteremia^j^	130 (3.4)	126 (3.5)	4 (2.4)	0.458

Urinary tract infection was defined as growth of bacteria at a concentration of at least 10^5^ CFU/mL in a midstream urine sample, 10^4^ CFU/mL in a catheter urine sample, or 10^3^ CFU/mL in a PCN urine sample or suprapubic urine sample. Only UTIs caused by a single strain of a uropathogen were included.

*CFU* colony-forming unit, *DMSA* Tc 99 m-dimercaptosuccinic acid, *ER* emergency room, *hsCRP* high-sensitivity C-reactive protein, *IQR* interquartile range, *PCN* percutaneous nephrostomy, *RBC* red blood cell, *SD* standard deviation, *UA* urinalysis, *UC* urine culture, *VCUG* voiding cystoureterography, *VUR* vesicoureteral reflux, *WBC* white blood cell.

^a^Categorical variables are presented as frequency (%) and continuous variables are presented as median (Q1, Q3), if not otherwise specified.

^b^To compare the difference between GPC and GNB, the *p* value was calculated using the chi-square test or Wilcoxon rank-sum test.

^c^Occurred any time prior to or on the day of the index UA order.

^d^Occurred within 1 year prior to or on the day of the index UA order.

^e^Occurred within 3 months prior to or on the day of the index UA order.

^f^The latest measure within 7 days prior to or on the day of the index UA order.

^g^Length of hospitalization was calculated for 939 patients (903 GN; 36 GP) who were hospitalized.

^h^Recurrent UTI was defined as having ≥ 2 UTIs within 6 months or having ≥ 3 UTIs within 1 year.

^i^Unilateral and bilateral ureteroneocystostomy that was performed anytime following the UA order.

^j^Bacteremia that occurred within 90 days following the UA order.

Compared with children with GN bacteria, those with GP bacteria were more likely to be older (≥ 24 months old; 29.4% vs 71.1%); be a girl (45.8% vs 60.8%); have a catheter, percutaneous nephrostomy, or suprapubic (33.2% vs 69.3%) urine specimen; or have prior admission (9.4% vs 13.3%; Table 1). By contrast, they were less likely to receive antibiotic prior to UTI (96.1% vs 91.0%), to have a Foley catheter in place (37.5% vs 12.7%), to have bacteremia (7.9% vs 3.0%), or to have fever (65.7% vs 31.1%). The serum biochemical profiles and urinalysis measures varied between GN and GP bacteria. Compared with patients with GN UTI, patients with GP UTI had higher serum hemoglobin (median, 11.2 vs 12.6 g/dL), lower serum WBC (14.6 vs 11.1 × 10^3^/μL), platelet (344 vs 272 × 10^3^/μL), hsCRP (3.5 vs 0.7 mg/dL), urine WBC (452 vs 96/μL), and urine RBC (22 vs 16/μL). Compared with that in GN UTI, urine bacteria (51.5% vs 27.7%), leukocyte esterase (92.4% vs 70.5%), and nitrite (38.8% vs 7.2%) were less likely to test positive in GP UTI. Patients with GP UTI were more likely to have a 1-day-shorter hospital stay and undergo ureteroneocystostomy (GN vs GP, 1.4% vs 3.6%). However, outcomes such as recurrent UTI, and bacteremia within 3 months following UA were similar between patients with GN UTI and GP UTI.

**Figure 2:**
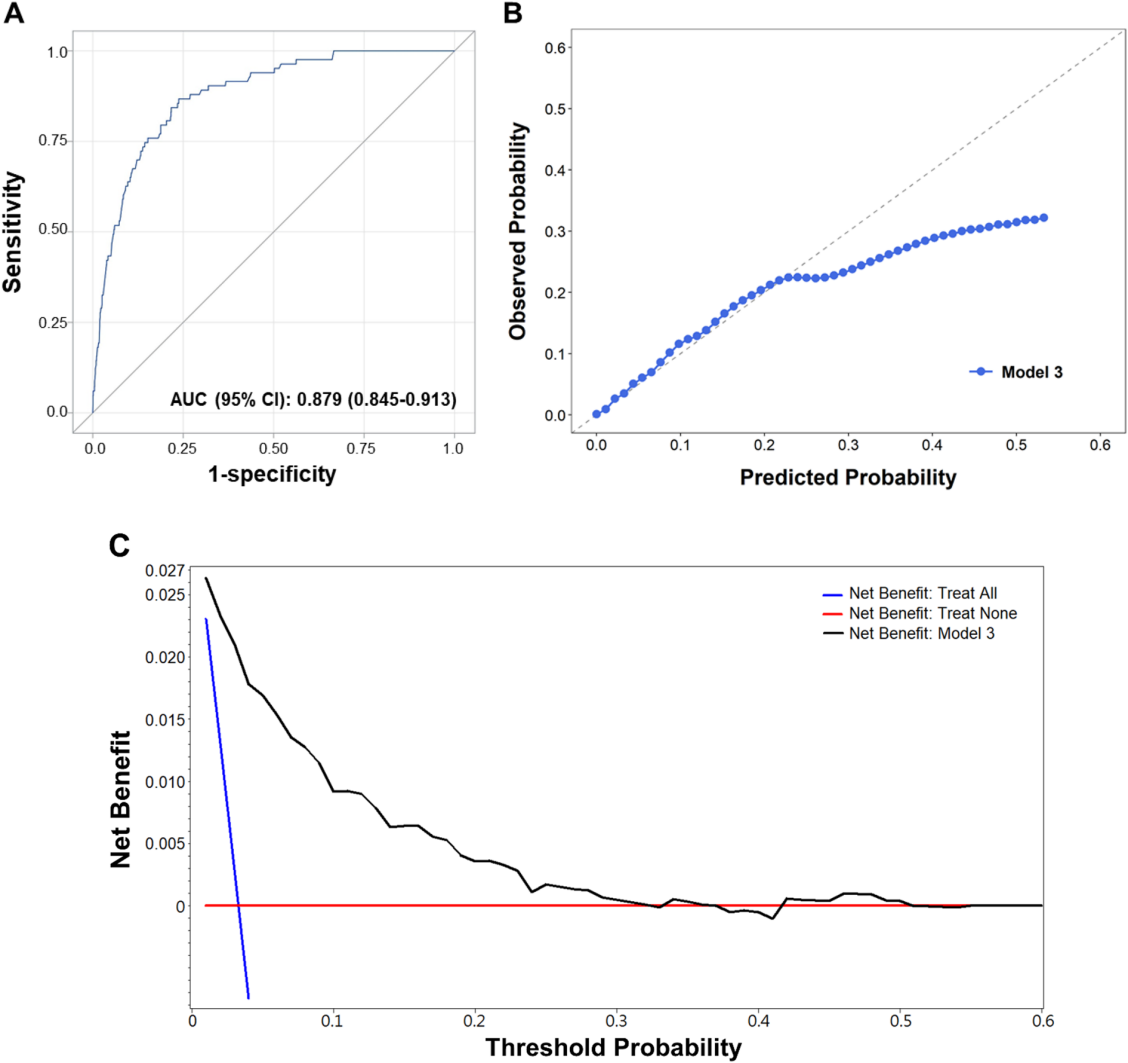
Discrimination plot (**A**), calibration plot (**B**), and decision curve (**C**) of the prediction model (Model 3) for pediatric urinary tract infection caused by gram-positive bacteria.

**Figure 3:**
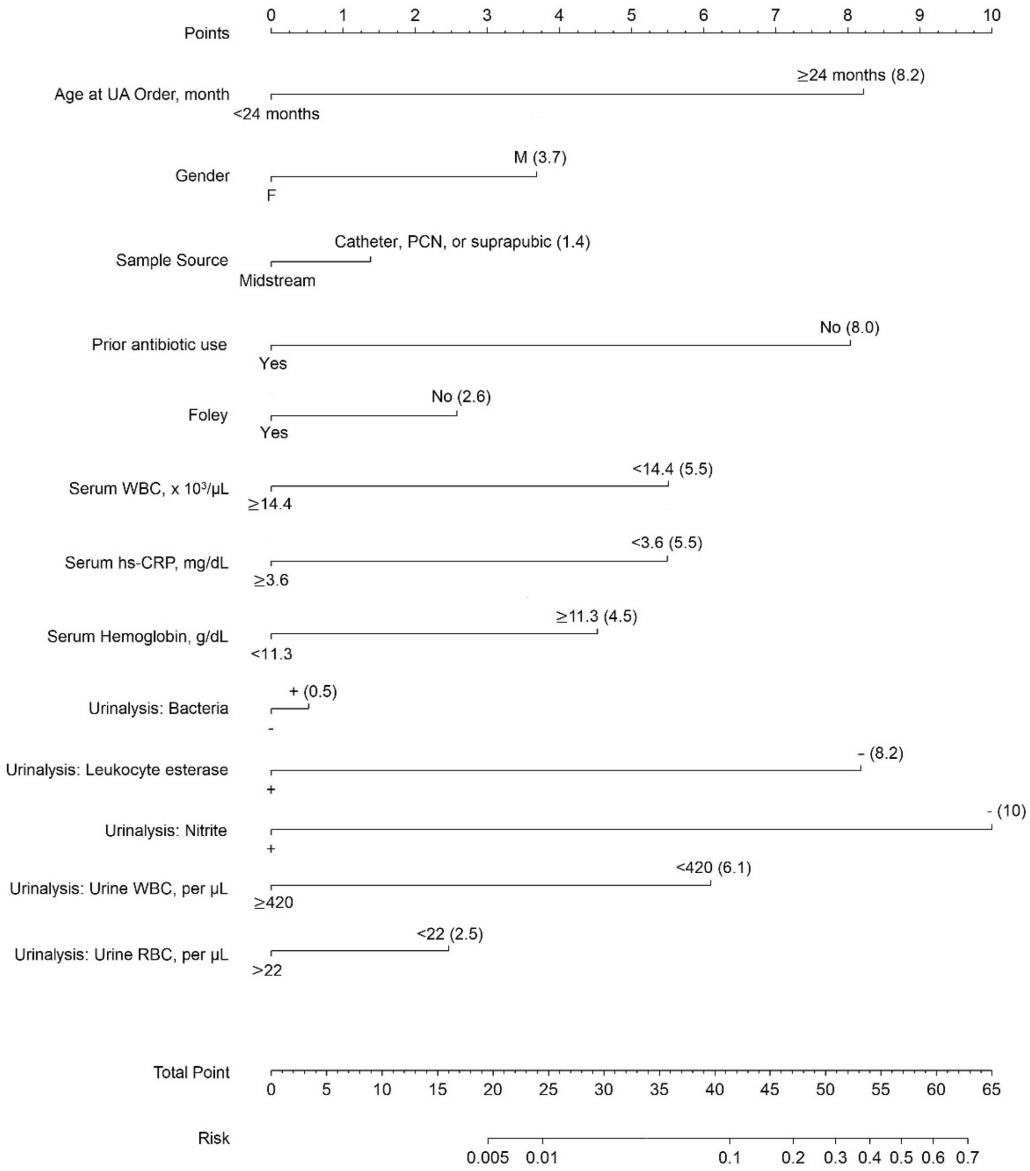
Nomogram of the prediction model for pediatric urinary tract infections caused by gram-positive bacteria.

Multivariable prediction model for GP UTI, including age ≥ 24 months; gender, boy; sampling source, catheter, PCN, or suprapubic; no history of antibiotic use; no Foley catheterization; serum WBC < 14.4 × 10^3^/μL; hsCRP < 3.4 mg/dL; hemoglobin ≥ 11.3 g/dL; presence of bacteria in urine; absence of leukocyte esterase or nitrite; urine WBC < 420/μL; and RBC < 22/μL, demonstrated good predictive performance (Model 3; c-statistic = 0.879; 95% CI 0.845–0.913; Table 2; Fig. 2A). Eight predictors, including age ≥ 24 months, no prior antibiotic use, decreased serum WBC, decreased hsCRP, increased hemoglobin, absence of leukocyte esterase or nitrite, and decreased urine WBC, were significantly associated with GP UTI. The model (Model 3) showed good calibration performance when the predictive probability was less than 25% (Fig. 2B), which suggests that the model provides a better net benefit and could improve clinical decision making (Fig. 2C). By using our nomogram, physicians can easily estimate the probability of UTI caused by a GP pathogen (Fig. 3).

**Table 2:** Multivariable logistic regression model for predicting pediatric urinary tract infections caused by gram-positive bacteria.

Variable	Crude OR	Model 1	Model 2	Model 3
	(95% CI)	(95% CI)	(95% CI)	(95% CI)
N	N = 3783	N = 3783	N = 2632	N = 2529
Age at UA order ≥ 24 months	5.90 (4.19, 8.31)	3.98 (2.25, 7.03)	3.01 (1.37, 6.63)	3.21 (1.31, 7.89)
Boy	0.54 (0.40, 0.75)	1.04 (0.73, 1.47)	1.71 (1.03, 2.82)	1.69 (0.97, 2.94)
Sample obtained from catheter, PCN, or suprapubic	4.54 (3.24, 6.36)	1.29 (0.75, 2.23)	1.21 (0.57, 2.55)	1.22 (0.52, 2.84)
No prior antibiotic use	2.43 (1.39, 4.24)	2.21 (1.24, 3.93)	3.20 (1.25, 8.21)	3.13 (1.12, 8.75)
No prior Foley catheterization	4.15 (2.61, 6.59)	1.70 (1.00, 2.89)	1.80 (0.94, 3.43)	1.44 (0.74, 2.83)
**Serum biochemical profiles**				
WBC < 14.4 × 10^3^/μL	3.96 (2.46, 6.38)	–	2.90 (1.70, 4.93)	2.19 (1.25, 3.83)
hsCRP < 3.4 mg/dL	3.33 (2.03, 5.48)	–	2.09 (1.23, 3.52)	2.18 (1.24, 3.85)
Hemoglobin ≥ 11.3 g/dL	4.54 (2.74, 7.50)	–	2.16 (1.19, 3.90)	1.90 (1.03, 3.51)
**Urinalysis**				
Bacteria+	0.35 (0.25, 0.50)	–	–	1.08 (0.63, 1.85)
Leukocyte esterase−	5.11 (3.58, 7.29)	–	–	3.19 (1.88, 5.42)
Nitrite−	8.15 (4.51, 14.73)	–	–	4.13 (1.87, 9.14)
WBC < 420/μL	3.45 (2.39, 4.96)	–	–	2.37 (1.18, 4.79)
RBC < 22/μL	1.38 (1.01, 1.88)	–	–	1.42 (0.82, 2.44)
C-statistic		0.745 (0.688, 0.801)	0.806 (0.753, 0.860)	0.879 (0.845, 0.913)

*CI* confidence interval, *hsCRP* high sensitivity C-reactive protein, *OR* odds ratio, *PCN* percutaneous nephrostomy, *RBC* red blood cell, *UA* urinalysis, *WBC* white blood cell.

## Discussion

In this 14-year hospital-based cohort study, we found that the distribution of GP UTI was stable over the study period, with GP bacteria contributing to approximately 4.4% of all pediatric UTI events. The top three pathogens for GN and GP uropathogens were *E. coli*, *P. mirabilis*, and *K. pneumoniae*, and vancomycin-resistant *E. faecalis* (VRE), *S. saprophyticus*, and coagulase-negative *Staphylococci*, respectively. VRE was the causative GP uropathogen in children younger than 2 and between 2 and 11 years of age; however, *S. saprophyticus* was predominant in children older than 12 years of age. Our prediction model for GP UTI in children has both good discrimination and calibration and the nomogram can make clinicians aware of the potential GP uropathogens.

The leading GP uropathogens and their distribution across age groups found in our study were consistent with that in the literature. *Enterococcus* spp. is the most common GP uropathogen in the pediatric outpatient population in the US^20^. *S. saprophyticus* is the most common (55.8%) UTI-causing GP bacteria in children ≥ 12 years old. *S. saprophyticus* is a common uropathogen in teenage girls or young adult women, especially in those with active sex lives. *S. saprophyticus* caused UTI in 24.5% female adolescents who visited the emergency department for UTI^21^. As *S. saprophyticus* is resistant to antibiotics used for the empirical treatment of UTI^22^, clinicians should be aware that *S. saprophyticus* could be responsible for the etiology of UTI in adolescents.

Our study demonstrated that VR *E. faecalis* accounted for 27.1% of pediatric GP UTI and caused more than half of GP UTI (54.2%) in children younger than 2 years. To our knowledge, few reports have discussed pediatric UTI caused by VRE^23^. VRE is a rapidly emerging multidrug-resistant pathogen causing infection in adults since its discovery in 1986^24^. A nationwide study of hospitalized children in the United States documented that VRE infection increased from 53 per million in 1997 to 120 per million in 2012^25^. As VRE poses a critical threat to hospital infection control, our findings facilitate risk management of pediatric UTI and inform infection control policy in children, especially those younger than 2 years of age, with GP UTI.

Marrow responses may help differentiate GP UTI from GN UTI. In our study, children with GP UTI had a lower WBC and platelet count and lower hsCRP level. This finding indicates that GP UTI has a lower inflammatory response, which is consistent with previous studies suggesting a more profoundly elevated WBC count and erythrocyte sedimentation rate in *E. coli* UTI compared with non-*E. coli* UTI in children^26,27^. The absence of urine nitrate or leukocyte esterase and the presence of pyuria indicated higher odds of GP UTI in our study and in two other studies^5,28^. Decreased levels of inflammatory biomarkers in the serum and urine in GP UTI may be because GP bacteria form biofilm-like intracellular bacterial communities within the epithelial cells lining the bladder lumen to avoid the host immune response^5^. Furthermore, unlike the enteric GN uropathogens, such as *E. coli*, that can efficiently reduce urinary nitrate to nitrite, most GP organisms, such as *Enterococcus* spp., *S. sprophyticus*, and group B *Streptococcus*, cannot^6,29^. Thus, urine nitrite can be used as a clinical marker to exclude enterococcal bacteriuria^28^.

The rate of ureteroneocystostomy, an operation to correct VUR, is higher in children with GP UTI. Pediatric non-*E. coli* UTI is associated with anatomical abnormalities, specifically VUR^26,27,30^. *Enterococcus*, *S. aureus*, and coagulase-negative *Staphylococci* are associated with VUR possibly because the urinary tract abnormalities allow low virulence GP bacteria to attach^9,10,30^. The common approaches to VUR include vigilant observation, antibiotic prophylaxis, and surgical correction, which is indicated in children with persistent VUR, recurrent UTI under antimicrobial prophylaxis, or high-grade reflux^15,31^.

Delayed treatment for febrile UTIs is significantly associated with permanent renal scarring^3^. Cefazolin, the first-line empirical therapy for UTIs, frequently fails to cover UTIs caused by GP bacteria and thus can increase the risk of renal scarring, especially among children with GP pyelonephritis^6,9,27,29,30^. Therefore, our prediction model for GP UTI can guide physicians to initiate appropriate antibiotic treatment early.

The strengths of our study include objective guideline-approved UTI definition using colony count and culture source, large sample size of 3783 pediatric UTIs, robust multivariable analysis to establish a prediction model for GP UTI, and addition of serum biomarkers to the prediction model for GP UTI. Our study also had a few limitations. First, our prediction model for GP UTI cannot be applied to UTIs with mixed GP and GN bacteria because our model was developed using GP- and GN-only UTIs. Second, some of the unmeasured features, such as history of UTI, prior antibiotic treatment, or other clinical histories, that were documented in other institutions, could have affected the likelihood of GP UTI. Third, our prediction model, developed in a tertiary medical center in central Taiwan, may not be applicable to other healthcare facilities. However, as one of the largest tertiary medical centers in Taiwan, our patient population should be representative.

This is the first study to establish a prediction model for GP UTI in a pediatric population. Age older than 2 years, no prior antibiotic use, low blood and urine WBC count, low hsCRP level, high hemoglobin level, and absence of urine nitrite and leukocyte esterase are significant predictors of pediatric UTI caused by GP bacteria. Our prediction model for GP UTI in children could help clinicians quantify the probability of infection by GP uropathogens and enable them to choose an adequate antibiotic regimen early. Large prospective studies in the future should validate our findings.

## Supplementary Information

Supplementary Figure 1.

Supplementary Figure 2.

Supplementary Figure 3.

Supplementary Figure 4.

Supplementary Table 1.

Supplementary Information.

## References

[1] Spencer JD, Schwaderer A, McHugh K, Hains DS (2010). Pediatric urinary tract infections: An analysis of hospitalizations, charges, and costs in the USA. Pediatr. Nephrol. (Berlin, Germany).

[2] Shortliffe LM, McCue JD (2002). Urinary tract infection at the age extremes: Pediatrics and geriatrics. Am. J. Med..

[3] Shaikh N (2016). Early antibiotic treatment for pediatric febrile urinary tract infection and renal scarring. JAMA Pediatr..

[4] Laupland KB, Ross T, Pitout JD, Church DL, Gregson DB (2007). Community-onset urinary tract infections: A population-based assessment. Infection.

[5] Shaikh N (2016). Association between uropathogen and pyuria. Pediatrics.

[6] Chaudhari PP, Monuteaux MC, Bachur RG (2017). Should the absence of urinary nitrite influence empiric antibiotics for urinary tract infection in young children?. Pediatr. Emerg. Care.

[7] Marcus N, Ashkenazi S, Samra Z, Cohen A, Livni G (2012). Community-acquired enterococcal urinary tract infections in hospitalized children. Pediatr. Nephrol. (Berlin, Germany).

[8] Bitsori M, Maraki S, Raissaki M, Bakantaki A, Galanakis E (2005). Community-acquired enterococcal urinary tract infections. Pediatr. Nephrol. (Berlin, Germany).

[9] Lubell TR (2016). Comparison of febrile infants with enterococcal and gram-negative urinary tract infections. Pediatr. Infect. Dis. J..

[10] Megged O (2014). *Staphylococcus aureus* urinary tract infections in children are associated with urinary tract abnormalities and vesico-ureteral reflux. Pediatr. Nephrol. (Berlin, Germany).

[11] Yeh HC (2020). 24-Hour serum creatinine variation associates with short- and long-term all-cause mortality: A real-world insight into early detection of acute kidney injury. Sci. Rep..

[12] Liang HY, Lo YC, Chiang HY, Chen MF, Kuo CC (2020). Validation and comparison of the 2003 and 2016 diastolic functional assessments for cardiovascular mortality in a large single-center cohort. J. Am. Soc. Echocardiogr..

[13] Lai HC (2019). Association between urine pH and common uropathogens in children with urinary tract infections. J. Microbiol. Immunol. Infect..

[14] Wu TY, Majeed A, Kuo KN (2010). An overview of the healthcare system in Taiwan. London J. Prim. Care (Abingdon).

[15] Stein R (2015). Urinary tract infections in children: EAU/ESPU guidelines. Eur. Urol..

[16] National Health Insurance Administration, Ministry of Health and Welfare, Taiwan. *Patients with catastrophic illnesses or rare diseases.* Online article at https://www.nhi.gov.tw/english/Content_List.aspx?n=F5B8E49CB4548C60&topn=1D1ECC54F86E9050 (2016).

[17] Alba AC (2017). Discrimination and calibration of clinical prediction models: Users' guides to the medical literature. JAMA.

[18] Harrell Jr, F. E. Regression Modeling Strategies. With Applications to Linear Models, Logistic and Ordinal Regression, and Survival Analysis. eBook ISBN 978-3-319-19424-0. Document of package 'rms' at https://cran.r-project.org/web/packages/rms/rms.pdf. (2021)

[19] Vickers AJ, van Calster B, Steyerberg EW (2019). A simple, step-by-step guide to interpreting decision curve analysis. Diagn. Progn. Res..

[20] Edlin RS, Shapiro DJ, Hersh AL, Copp HL (2013). Antibiotic resistance patterns of outpatient pediatric urinary tract infections. J. Urol..

[21] Lo DS, Shieh HH, Barreira ER, Ragazzi SL, Gilio AE (2015). High frequency of staphylococcus saprophyticus urinary tract infections among female adolescents. Pediatr. Infect. Dis. J..

[22] Pailhories H (2017). *Staphylococcus saprophyticus*: Which beta-lactam?. Int. J. Infect. Dis..

[23] Shrestha LB, Baral R, Poudel P, Khanal B (2019). Clinical, etiological and antimicrobial susceptibility profile of pediatric urinary tract infections in a tertiary care hospital of Nepal. BMC Pediatr..

[24] O'Driscoll T, Crank CW (2015). Vancomycin-resistant enterococcal infections: Epidemiology, clinical manifestations, and optimal management. Infect. Drug Resist..

[25] Adams DJ, Eberly MD, Goudie A, Nylund CM (2016). Rising vancomycin-resistant enterococcus infections in hospitalized children in the United States. Hosp. Pediatr..

[26] Shaikh N (2016). Predictors of non-*Escherichia coli* urinary tract infection. Pediatr. Infect. Dis. J..

[27] Friedman S, Reif S, Assia A, Mishaal R, Levy I (2006). Clinical and laboratory characteristics of non-*E. coli* urinary tract infections. Arch. Disease Childhood.

[28] Holloway J, Joshi N, O'Bryan T (2000). Positive urine nitrite test: An accurate predictor of absence of pure enterococcal bacteriuria. South Med. J..

[29] Kline KA, Lewis AL (2016). Gram-positive uropathogens, polymicrobial urinary tract infection, and the emerging microbiota of the urinary tract. Microbiol. Spectr..

[30] Honkinen O, Lehtonen OP, Ruuskanen O, Huovinen P, Mertsola J (1999). Cohort study of bacterial species causing urinary tract infection and urinary tract abnormalities in children. BMJ (Clin. Res. Ed.).

[31] Tekgül S (2012). EAU guidelines on vesicoureteral reflux in children. Eur. Urol..

