# Supplementary material for: Clinical characteristics and prediction analysis of pediatric urinary tract infections caused by gram-positive bacteria
Source: Sci Rep. 2021 May 26;11:11010. doi: 10.1038/s41598-021-90535-6 (PMC8155007; doi:10.1038/s41598-021-90535-6)
Supplement: Supplementary file 3 — Supplementary Figure 3. [file 41598_2021_90535_MOESM3_ESM.docx]

**Supplemental Figure 3**. Distribution of gram-negative bacteria that cause pediatric urinary tract infections (N = 3,617 patients).
