# Supplementary material for: Clinical characteristics and prediction analysis of pediatric urinary tract infections caused by gram-positive bacteria
Source: Sci Rep. 2021 May 26;11:11010. doi: 10.1038/s41598-021-90535-6 (PMC8155007; doi:10.1038/s41598-021-90535-6)
Supplement: Supplementary file 4 — Supplementary Figure 4. [file 41598_2021_90535_MOESM4_ESM.docx]

**Supplemental Figure 4**. Distribution of gram-positive bacteria that cause pediatric urinary tract infections, by age group.
