# Supplementary material for: Clinical characteristics and prediction analysis of pediatric urinary tract infections caused by gram-positive bacteria
Source: Sci Rep. 2021 May 26;11:11010. doi: 10.1038/s41598-021-90535-6 (PMC8155007; doi:10.1038/s41598-021-90535-6)
Supplement: Supplementary file 5 — Supplementary Table 1. [file 41598_2021_90535_MOESM5_ESM.docx]

**Supplemental Table 1.** Antimicrobial treatment received within 7 days of urinalysis for 3,783 pediatric patients with urinary tract infection.

| **Antimicrobial Agent** | **Gram-Negative**  **(N = 3,617)** |  | **Gram-Positive**  **(N = 166)** | **p-value** |
| --- | --- | --- | --- | --- |
|  | **N (Col %)** |  | **N (Col %)** |  |
| Cephalosporin 1^st^ generation | 3217 (88.9) |  | 117 (70.5) | <0.0001 |
| Cefazolin | 2569 (71.0) |  | 61 (36.7) | <0.0001 |
| Cefadroxil Susp | 55 (1.52) |  | 1 (0.60) | 0.52 |
| Cephalexin Susp | 209 (5.78) |  | 7 (4.21) | 0.49 |
| Cephalexin | 3 (0.08) |  | 2 (1.20) | 0.02 |
| Cephradine | 381 (10.5) |  | 46 (27.7) | <0.0001 |
| Cephalosporin 2^nd^ generation | 71 (1.96) |  | 1 (0.60) | 0.37 |
| Cephalosporin 3^rd^ generation | 430 (11.9) |  | 15 (9.04) | 0.26 |
| Cephalosporin 4^th^ generation | 17 (0.47) |  | 1 (0.60) | 0.55 |
| Amphenicols | 1 (0.03) |  | 0 | >0.99 |
| Aminoglycosides | 0 |  | 0 | - |
| Beta-lactamase inhibitors | 0 |  | 0 | - |
| Beta-lactamase resistant penicillins | 3 (0.08) |  | 0 | >0.99 |
| Beta-lactamase sensitive penicillins | 7 (0.19) |  | 2 (1.20) | 0.06 |
| Carbapenems | 124 (3.42) |  | 1 (0.60) | 0.04 |
| Combinations of penicillins, incl. beta-lactamase inhibitors | 168 (4.64) |  | 16 (9.64) | 0.003 |
| Fluoroquinolones | 46 (1.27) |  | 2 (1.20) | >0.99 |
| Glycopeptide antibacterials | 20 (0.55) |  | 3 (1.81) | 0.08 |
| Imidazole derivatives | 7 (0.19) |  | 0 | >0.99 |
| Intermediate-acting sulfonamides | 1 (0.03) |  | 0 | >0.99 |
| Lincosamides | 3 (0.08) |  | 0 | >0.99 |
| Macrolides | 21 (0.58) |  | 3 (1.81) | 0.09 |
| Monobactams | 0 |  | 0 | - |
| Other aminoglycosides | 1855 (51.3) |  | 43 (25.9) | <0.0001 |
| Amikacin sulfate | 18 (0.50) |  | 0 | 0.36 |
| Gentamycin | 1837 (50.8) |  | 43 (25.9) | <0.0001 |
| Other antibacterials | 427 (11.8) |  | 26 (15.7) | 0.13 |
| Penicillins with extended spectrum | 220 (6.08) |  | 27 (16.3) | <0.0001 |
| Polymyxins | 0 |  | 1 (0.60) | 0.04 |
| Steroid antibacterials | 1 (0.03) |  | 1 (0.60) | 0.09 |
| Tetracyclines | 1 (0.03) |  | 0 | >0.99 |
