# Supplementary material for: Clinical characteristics and prediction analysis of pediatric urinary tract infections caused by gram-positive bacteria
Source: Sci Rep. 2021 May 26;11:11010. doi: 10.1038/s41598-021-90535-6 (PMC8155007; doi:10.1038/s41598-021-90535-6)
Supplement: Supplementary file 6 — Supplementary Information. [file 41598_2021_90535_MOESM6_ESM.docx]

**Supplemental Text.** Urinalysis and Urine Culture Methods

The urine routine biochemistry analysis was performed for glucose, bilirubin, ketone, specific gravity, occult blood, pH, protein, urobilinogen, nitrite, and leukocyte esterase by using the CLINITEK Atlas reagent and CLINITEK Automated Urine Chemistry Analyzer (SIEMENS Healthcare, Huizingen, Belgium) before January 14, 2014, and by using the AUTION Sticks 10EA reagent and Urine Analysis System AUTION AE-4030 (ARKRAY Factory, Inc., Shiga, Japan) after January 15, 2014. Microscopic examination of urine WBC, red blood cell (RBC), and epithelial cells was also performed.

Urine specimens from midstream urine, urine catheter, urine percutaneous nephrostomy (PCN), or urine suprapubic were inoculated using 0.001 mL inoculation loop on trypticase soy agar with 5% sheep blood and eosin methylene blue agar and cultured at 35°C for at least 16 h. CFU per mL were then calculated by multiplying colony counts by 1,000. Bacterial species were identified using MALDI Biotyper System (Bruker Daltonik GmbH, Bremen, Germany), since 2014. Antimicrobial susceptibility testing (AST) results were analyzed using Pheonix^TM^ Automated Microbiology System (BD Diagnostics, Sparks, MD, USA), since 2006. Prior to the implementation of the automatic system, bacterial species and AST were identified using traditional methods following the Clinical and Laboratory Standards Institute guideline (https://clsi.org/standards/).
